# Novel characteristics for immunophenotype, FISH pattern and molecular cytogenetics in synovial sarcoma

**DOI:** 10.1038/s41598-023-34983-2

**Published:** 2023-05-16

**Authors:** Ling ling Zhong, Gao xiang Huang, Li ying Xian, Zong chen Wei, Zhi ping Tang, Qiu yue Chen, Hao Chen, Fang Tang

**Affiliations:** 1 Department of Pathology, The 924th Hospital of the Chinese People’s Liberation Army Joint Logistic Support Force, Guangxi Key Laboratory of Metabolic Diseases Research, Guilin, 541002 Guangxi China; 2 Guangxi Key Laboratory of Glucose and Lipid Metabolic Diseases, The Second Affiliated Hospital of Guilin Medical University, Guilin, 541199 Guangxi China; 3 Department of Pathology and Laboratory Medicine, Dongguan Affiliated Hospital of Southern Medical University, Dongguan, 523059 Guangdong China

**Keywords:** Surgical oncology, Molecular medicine

## Abstract

As a rare and highly aggressive soft tissue sarcoma, the new immunophenotype, atypical FISH pattern and relevant molecular cytogenetics of synovial sarcoma (SS) remain less known, although it is characteristically represented by a pathognomonic chromosomal translocation t (X; 18) (p11.2; q11.2). Methodologically, the morphology was retrospectively analysed by using H&E staining, and immunohistochemical features were investigated by using markers that have been recently applied in other soft tissue tumors. Moreover, FISH signals for *SS18* and *EWSR-1* break-apart probes were examined. Finally, cytogenetic characteristics were analysed via RT-PCR and Sanger sequencing. Consequently, nine out of thirteen cases that were histologically highly suspected as SS were finally identified as SS via molecular analysis. Histologically, nine SS cases were divided into monophasic fibrous SS (4/9), biphasic SS (4/9) and poorly differentiated SS (1/9). Immunohistochemically, SOX-2 immunostaining was positive in eight cases (8/9) and PAX-7 immunostaining was diffusely positive in the epithelial component of biphasic SS (4/4). Nine cases showed negative immunostaining for NKX3.1 and reduced or absent immunostaining for INI-1. Eight cases showed typically positive FISH signalling for the *SS18* break-apart probe, whereas one case exhibited an atypical FISH pattern (complete loss of green signalling, case 2). Furthermore, the *SS18-SSX1* and *SS18-SSX2* fusion genes were identified in seven cases and two cases, respectively. The fusion site in 8 out of 9 cases was common in the literature, whereas the fusion site in case 2 was involved in exon 10 codon 404 in *SS18* and exon 7 codon 119 in *SSX1* (which has not been previously reported), which notably corresponded to the complete loss of green signalling in the FISH pattern. Additionally, FISH analysis of the *EWSR-1* gene in nine SS cases demonstrated aberrant signalling in three cases that were recognized as a monoallelic loss of *EWSR-1* (1/9), an amplification of *EWSR-1* (1/9) and a translocation of *EWSR-1* (1/9). In conclusion, *SS18-SSX* fusion gene sequencing is obligatory for a precise diagnosis of SS when dealing with a confusing immunophenotype and atypical or aberrant FISH signalling for *SS18* and *EWSR-1* detection.

## Introduction

Synovial sarcoma (SS) is a less common and highly aggressive soft tissue sarcoma and accounts for approximately 8–10% of soft tissue sarcomas^1–3^. Although SS may occur at any site throughout the body and at any age, it has a propensity to affect the extremities and to affect adolescents and young adults. Morphologically, SS is a heterogeneous tumor and mainly forms three histological variants, including monophasic SS, biphasic SS and poorly differentiated SS^2^. Immunohistochemically, in addition to conventional markers such as EMA, AE1/AE3, Bcl-2 and CD99, TLE-1 has been previously reported to be a sensitive marker for SS diagnosis^4,5^. Decreased INI-1 expression has also been observed in more than 80% of SS cases in two previous studies, in comparison with the complete loss of expression in INI-1-deficient neoplasm mimics, such as epithelioid sarcoma and malignant rhabdoid tumors^6,7^. Recently, SS18-SSX fusion-specific antibodies have been implicated in diagnosing SS and shown to correlate quite well with the fusion gene status^8,9^.

More than 95% of SS cases harbour the unique pathognomonic translocation t(X;18) (p11.2; q11.2), thus resulting in the *SS18-SSX* fusion gene. Among these SS cases, approximately two-thirds of the cases harbour the *SS18-SSX1* fusion gene, one-third of the cases harbour the *SS18-SSX2* fusion gene and rare SS cases harbour the *SS18-SSX4* fusion gene^10,11^. Only one SS case has been reported to be characterized by the t(X;20) translocation, thus resulting in the *SS18L1-SSX1* fusion gene^12^. Thus, molecular detection methods, such as FISH and RT-PCR, are valuable tools in the identification of SS.

Although SS has been well studied in clinical, morphological, immunohistochemical and cytogenetic aspects, new immunostaining markers, atypical FISH patterns and accompanying molecular alterations in SS are still less known. In this study, we investigated the expression of new immunohistochemical markers, the FISH pattern, the fusion gene sequence and accompanying gene changes in nine SS cases that were finally identified by using molecular detection. We found that a panel of immunostaining markers, including TLE-1, SOX-2, PAX-7, INI-1 and NKX3.1, may be useful ancillary tools in SS diagnosis. With regard to the FISH assay, the complete loss of green signal, an atypical or abnormal FISH pattern with the SS18 break-apart probe, and a new fusion gene sequence were observed in one SS case for the first time. Furthermore, *EWSR-1* gene changes were observed in a minority of SS cases.

## Methods

### Case selection

Thirteen cases that were morphologically highly suspected as synovial sarcoma were collected from the Department of Pathology, the PLA Joint Logistic Support Force No. 924 Hospital (Guilin, Guangxi, China) and Dongguan Affiliated Hospital of Southern Medical University (Dongguan, Guangdong, China) during the time period from 2010 to 2019. Thirteen surgical resection specimens fixed in neutral buffered 10% formalin and embedded in paraffin were subjected to FISH, RT-PCR detection and gene sequence analysis procedures. Additionally, 9 out of 13 cases were genetically confirmed as being synovial sarcoma, whereas four cases failed regarding FISH analysis and *SS18-SSX* fusion transcript detection (due to poor nucleic acid quality). The present study was approved by the Ethics Committee of PLA Joint Logistic Support Force No. 924 Hospital and Dongguan Affiliated Hospital of Southern Medical University. Written informed consent was obtained from all of the patients or legal guardians.

### Histological evaluation

The 3-μm tissue sections from the formalin-fixed and paraffin-embedded (FFPE) tumor specimens were stained by using routine haematoxylin and eosin (H&E). The H&E-stained slides were re-reviewed by three surgical pathologists (LLZ, GXH and FT) under a multi-head microscope and preliminary diagnoses were confirmed. Moreover, the histological subtypes of synovial sarcoma were divided into monophasic, biphasic and poorly differentiated types based on the criteria of Soft Tissue and Bone Tumors, WHO Classification of Tumors, 5th Edition^2^. Tumor grade was determined by using the Fédération Nationale des Centres de Lutte contre le Cancer (FNCLCC) grading system^13^.

### Immunohistochemistry

The 3-μm whole tissue sections from FFPE tumor specimens with both negative and positive controls were automatically retrieved and immunostained using the EnVision technique in the Ventana BenchMark XT instrument (Ventana Medical Systems, Tucson, AZ, USA), followed by a light haematoxylin counterstain. For the indicated antibodies, the procedure of antigen heat retrieval was 100 °C for 30 min by using the EDTA buffer (pH = 8.4). The procedure of primary antibody incubation was 37 °C for 1 h. Information on commercially available antibodies against cytokeratin (AE1/AE3), EMA, CD99, Bcl-2, TLE1, NKX3.1, SOX2, PAX-7 and INI-1 was listed in Table 1. In principle immunohistochemical staining was independently evaluated by two investigators (LLZ and GXH). In the face of inconsistency between investigators, all investigators in this project would be requested to evaluate and discuss until a consensus was reached. Furthermore, the intensity of staining was scored as “-” (negative), “1+” (weak positive), “2+” (moderate positive) or “3+” (strong positive). The extent of staining was evaluated as focal (< 10% of tumor cells), diffuse (> 75% of tumor cells) or specific percentage (approximately 10–75% of tumor cells).

**Table 1:** Antibodies that were used in this study and subcellular distribution.

Antibody	Corporation	Clone	Dilution	Subcellular distribution
Cytokaretin (CK-pan)	ZSGB-BIO	Monoclonal AE1/AE3	Ready to use	Cytoplasm/membrane
EMA	ZSGB-BIO	Monoclonal GP1.4	Ready to use	Cytoplasm/membrane
CD99	ZSGB-BIO	Monoclonal EP8	Ready to use	Cytoplasm/membrane
Bcl-2	ZSGB-BIO	Monoclonal EP36	Ready to use	Cytoplasm/membrane
TLE1	ZSGB-BIO	Monoclonal UMAB253	Ready to use	Nucleus
NKX3.1	ZSGB-BIO	Polyclonal	Ready to use	Nucleus
SOX2	ZSGB-BIO	monoclonal EP103	Ready to use	Nucleus
PAX7	Developmental Studies Hybridoma Bank	Monoclonal	1:200	Nucleus
INI-1	ZSGB-BIO	Monoclonal	Ready to use	Nucleus

### Fluorescence in situ hybridization (FISH)

Commercially available *SS18* and *EWSR-1* Break Apart Rearrangement Probes (cat#F.01083 and cat#F.01194) were purchased from Guangzhou Lbp Medicine Science & Technology Co. (Guangzhou, Guangdong, China). For the *SS18* break-apart probe, one end of the probe was labelled with the red spectrum (telomeric, 5′ to *SS18*, 649 kb), and the other end was labelled with the green spectrum (centromeric, 3′ to *SS18*, 925 kb); additionally, the probes were separated by a gap of 131 kb within the *SS18* gene. For the *EWSR-1* break apart probe, one end of the probe was labelled with the green spectrum (telomeric, 5′ to *EWSR-1*, 826 kb), and the other end was labelled with the red spectrum (centromeric, 3′ to *EWSR-1*, 439 kb); additionally, the probes were separated by a gap of 152 kb within the *EWSR-1* gene. FISH analysis was performed according to the manufacturer’s protocol. FISH assays were performed on 3-μm-thick FFPE tissue sections. The sections were deparaffinized in xylene twice for 30 min and dehydrated in 100% ethanol twice for 5 min. Additionally, the tissue sections were pretreated with high temperature and high pressure, after which they were digested with pepsin solution (4.0 mol/ml). After washing, tissue sections were denatured at 85 °C and hybridized with the indicated probe overnight at 37 °C in a humidified chamber. The slides were then washed with 2 × SSC at 37 °C for 10 min and again washed with 0.1% NP40/2 × SSC at room temperature for 5 min, followed by dehydrated using graded ethanol. Tissue slides were counterstained with 2.0 μg/ml DAPI. Fifty nonoverlapping tumor nuclei (which were clearly identified and contained unequivocal signals) were counted for each case. A split signal was considered positive for gene rearrangement if the distance between the green and red signals was greater than 2 signal diameters. Moreover, according to our laboratory practice experience and previous studies^14,15^, a case was considered positive for gene rearrangement when at least 15% of the tumor cells exhibited split-apart signals. In principle counting of the cases was independently performed by two investigators (LLZ and ZCW). Furthermore, the way to resolve the inconsistency between investigators was the same as that under immunohistochemical evaluation.

### Reverse transcription polymerase chain reaction (RT-PCR)

All tumor specimens with enough material were analysed for the presence of the fusion genes *SS18-SSX1*, *SS18-SSX2* or *SS18-SSX4* by using RT-PCR. Total RNA was isolated from two 10-μm tissue sections of FFPE specimen blocks by using the RNeasy FFPE Kit (Qiagen, Valencia, CA) according to the manufacturer’s instructions. To eliminate the contamination of genomic DNA, RNA samples were treated with DNase I. RNA reverse-transcription into cDNA was performed by using the Thermo RevertAid™ First Strand cDNA Synthesis Kit (Thermo Fisher Scientific Inc.) for 1 h at 42 °C and 5 min at 70 °C by using SSX-1/2-B reverse primer: 5′-cattttgtgggccagatgc-3′, as has been previously described^10^. Moreover, PCRs were performed for 35 cycles by using a QIAGEN Multiplex PCR Kit (Qiagen, Valencia, CA) with the following cycle conditions: denaturation at 94 °C for 30 s, annealing at 58 °C for 90 s and extension at 72 °C for 90 s. The primers were used in the following combinations: *SS18-SSX* consensus forwards primer: 5′-agaccaacacagcctggaccac-3′; *SS18-SSX1*-specific reverse primer: 5′-acactcccttcgaatcattttcg-3′; *SS18-SSX2*-specific reverse primer: 5′-gcacttcctccgaatcatttc-3′; and *SS18-SSX4*-specific reverse primer: 5′-gcacttccttcaaaccattttct-3′. cDNA from angiomatoid fibrous histiocytoma tissue was used as the negative control. Furthermore, glyceraldehyde-3-phosphate dehydrogenase (GAPDH) was used as the reference gene, and human *GAPDH* primers were obtained from Sangon Biotech Shanghai Co. Ltd. (Cat no. B661104). The products of the classic fusion gene and human *GAPDH* reference gene were 111 bp and 138 bp, respectively. The RT‒PCR products were further fractionated on 3.0% agarose gels and visualized via GoldView staining and ultraviolet illumination.

### Sequence analysis

PCR products were subjected to Sanger sequencing by Sangon Biotech Shanghai Co. Ltd. To accurately sequence the whole fragments, all of the PCR products were purified and then subjected to TA cloning. The obtained sequence data were < 200 bp and were analysed via online BLAST software (http://blast.ncbi.nlm.nih.gov/Blast.cgi).

### Ethics approval and consent to participate

The present study was approved by the ethics committee of PLA joint logistic support force No. 924 hospital (reference number: 201901106) and Dongguan Affiliated Hospital of Southern Medical University (reference number: 20190219). All methods were carried out in accordance with relevant guidelines and regulations. Informed consents were obtained from all patients or legal guardian for study participation.

## Results

### Clinical characteristics

The clinical information of the 9 patients is summarized in Table 2. In this case series, the ages of the patients ranged from 11 to 65 years old (median 45 years), and males were slightly more predominant than females (6:3). The maximum tumor diameter ranged from 1.2 to 8 cm (median 5 cm). Additionally, tumors were located in joints or near the joints in 6 cases and other uncommon sites in 3 cases, including the neck, abdomen and thigh. All of the patients received surgical tumorectomy, and 6 patients subsequently received adjuvant chemotherapy and/or radiotherapy. Follow-up information was available for 6 patients and the follow-up time ranged from 13 to 82 months (median 37 months). Lung metastasis occurred in 2 patients, and they died of disease within 23–35 months of initial diagnosis. Moreover, recurrence was identified at 24 months in 1 case; however, the patient was still alive with disease at 44 months. Three patients had no evidence of disease at 13, 39 and 82 months.

**Table 2:** The clinical information of nine patients who were genetically diagnosed as SS.

Case	Age/gender	Tumor size (cm)	Tumor location	Initial treatment	Recurrence/metastasis	Follow-up
1	32/F	5	Right planta	Tumorectomy	No	NED/39 m
2	11/F	1.2	Left juxta-articular of knee	Tumorectomy	NA	NA
3	48/M	7	Left toe	Tumorectomy + chemotherapy	No	NED/13 m
4	45/M	3.8	Right juxta-articular of knee	Tumorectomy + chemotherapy	NA	NA
5	65/M	6	Right elbow	Tumorectomy + chemotherapy	Lung metastasis/14 m	DOD/23 m
6	60/M	2	Left ankle	Tumorectomy + radiotherapy	NA	NA
7	42/F	5.5	Abdomen	Extended tumorectomy + radiotherapy	No	NED/82 m
8	42/M	2	Neck	Tumorectomy	Recurrence/24 m	AWD/44 m
9	58/M	8	Left thigh	Extended tumorectomy + radiotherapy + chemotherapy	Lung metastasis/10 m	DOD/35 m

*SS* synovial sarcoma, *F* female, *M* male, *NA* not available, *NED* no evidence of disease, *DOD* died of disease, *AWD* alive with disease.

### Histopathological features

**Figure 1:**
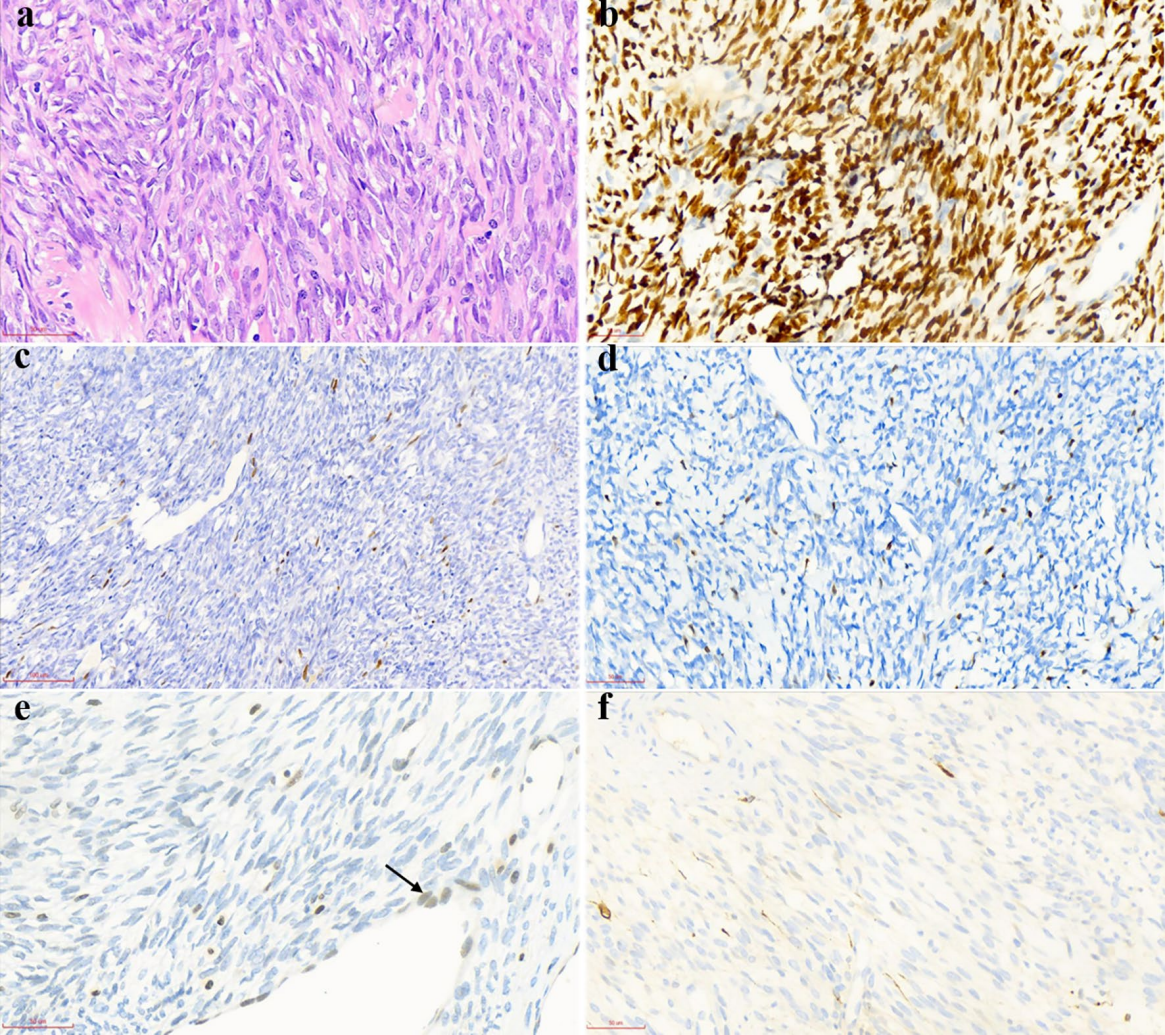
Morphological and immunohistochemical features in case 1. (**a**) Monophasic fibrous synovial sarcoma consists of uniform and delicate spindle tumor cells arranged in vague fascicles with hyalinized collagen (H&E, × 400). (**b**) TLE-1 immunostaining is diffusely and strongly positive in tumor cells (× 400). (**c**) SOX-2 immunostaining is focally and moderately positive in tumor cells (× 200). (**d**) PAX-7 immunostaining is focally and moderately positive in tumor cells (× 400). (**e**) INI-1 immunostaining is weak to absent in the majority of tumor cells. The vascular endothelial cells retain INI-1 expression as an internal control (arrowhead, × 400). (**f**) CK-pan immunostaining is focally and weakly positive in individual tumor cells (× 400).

**Figure 2:**
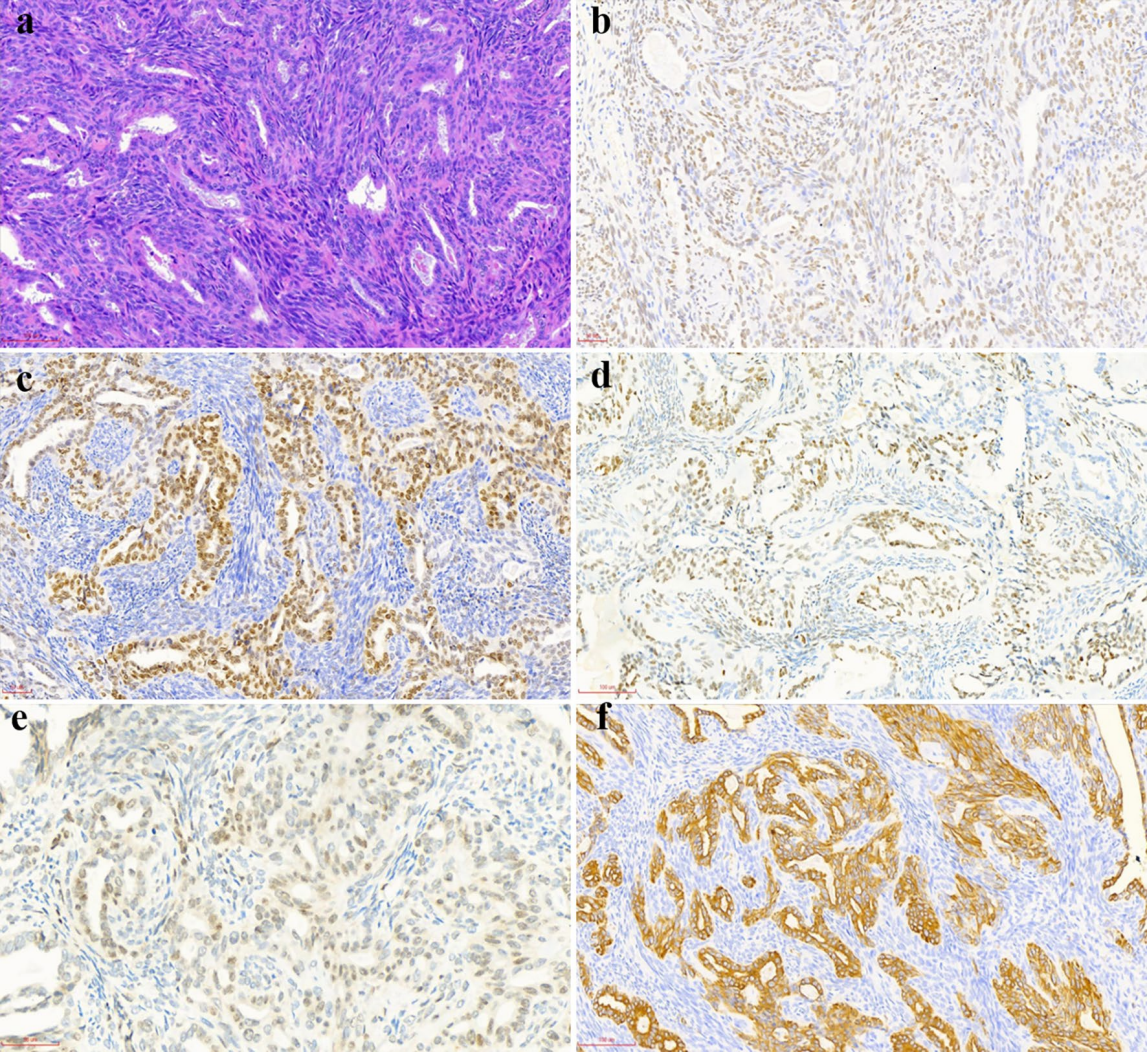
Morphological and immunohistochemical features in case 4. (**a**) Biphasic synovial sarcoma consists of a glandular epithelial tumor component and spindle tumor cells (H&E, × 200). (**b**) TLE-1 immunostaining is diffusely and moderately positive in epithelial and spindle components (× 200). (**c**) SOX-2 immunostaining is diffusely and strongly positive in the glandular tumor component but focally and weakly positive in the spindle tumor component (× 200). (**d**) PAX-7 immunostaining is diffusely and moderately positive in the glandular epithelial component but negative in the spindle tumor cells (× 200). (**e**) INI-1 retains moderate expression in the glandular epithelial component, but shows loss of expression in the majority of spindle tumor cells (× 400). (**f**) CK-pan immunostaining is diffusely and strongly positive in the glandular epithelial tumor component but negative in the spindle tumor component (× 200).

**Figure 3:**
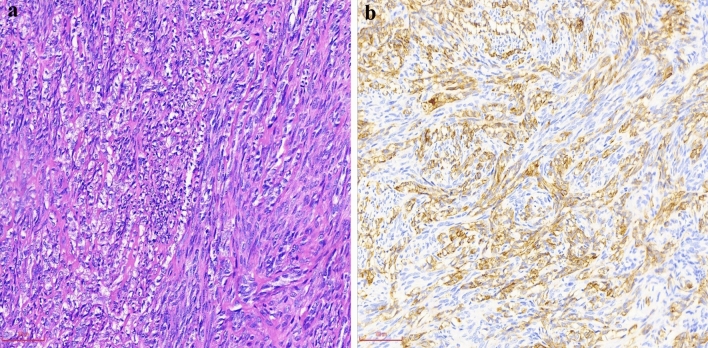
Morphological and immunohistochemical features in case 6. (**a**) Biphasic synovial sarcoma consists of mixed epithelial and spindle tumor components. The epithelial tumor cells have clear cytoplasm and are arranged in cords and nests (H&E, × 200). (**b**) CK-pan immunostaining is diffusely and strongly positive in the epithelial component but weakly positive in individual spindle tumor cells (× 200).

**Figure 4:**
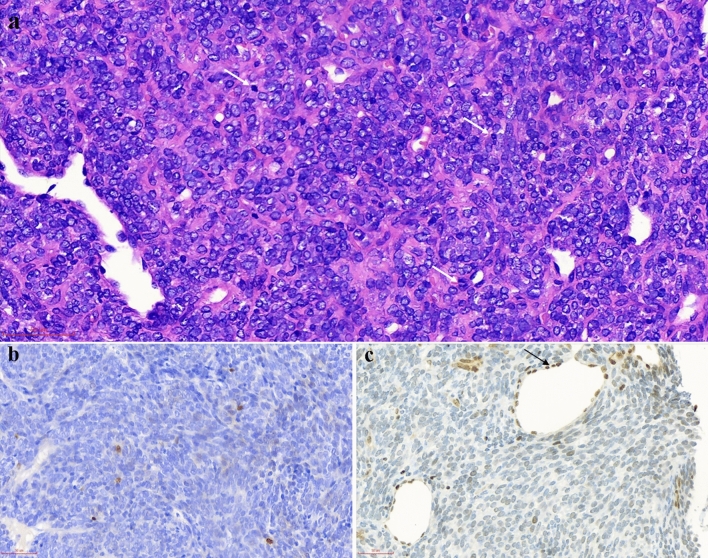
Morphological and immunohistochemical features in case 3. (**a**) Poorly differentiated synovial sarcoma consists of large epithelioid tumor cells with a haemangiopericytoma-like pattern, and the mitotic figures are easily visible (white arrowhead, H&E, × 400). (**b**) SOX-2 immunostaining is weakly positive in individual tumor cells (× 400). (**c**) INI-1 immunostaining is reduced in the majority of tumor cells compared with strong positivity in vascular endothelial cells (black arrowhead, × 400).

On the basis of morphological features of synovial sarcoma, we classified the tumor as monophasic fibrous synovial sarcoma (MFSS) (4/9, 44.5%), biphasic synovial sarcoma (BSS) (4/9, 44.5%) and poorly differentiated synovial sarcoma (PDSS) (1/9, 11%). The histological features of the nine cases are summarized in Table 3. MFSS is commonly composed of uniform and delicate spindle tumor cells with fascicle or dense sheet patterns. Moreover, spindle tumor cells often have sparse cytoplasm and hyperchromatic nuclei with inconspicuous nucleoli. Variable amounts of hyalinized collagen were observed in the tumor stroma (Fig. 1a). BSS was composed of mixed spindle and epithelial tumor cells (Figs. 2a, 3a). The spindle components resembled those of MFSS. Furthermore, the epithelial tumor cells were often arranged in glandular, tubular, nest or cord patterns, with occasional alveolar or papillary architecture. In the glandular area, the epithelial tumor cells were cuboidal and had ovoid nuclei and pale eosinophilic cytoplasm with intraluminal secretions (Fig. 2a). In the solid cord area, the epithelial tumor cells exhibited clear cytoplasm and a vague transition to spindle cells (Fig. 3a). PDSS was consistently composed of large epithelial cells with staghorn-shaped vessels. Furthermore, the epithelial cells had round or ovoid vesicular nuclei and were arranged in a sheet pattern. Mitotic figures were brisk in PDSS (Fig. 4a).

**Table 3:** The histopathological and immunohistochemical features of nine patients who were genetically diagnosed as SS.

Case	Morphology	Grade*	Mitotic rate/10 HPF	TLE-1	SOX-2	PAX-7	NKX3.1	INI-1	CK-pan	EMA	CD99	BCL-2
1	MFSS	2	3	D, 3 +	F, 2 +	F, 2 +	−	20%, 1 +	F, 1 +	20%, 2 +	D, 1 +	D, 2 +
2	MFSS	2	9	D, 3 +	F, 2 +	−	−	ND	20%, 3 +	30%, 2 +	D, 2 +	D, 2 +
3	PDSS	3	14	D, 2 +	F, 1 +	−	−	D, 1 +	F, 2 +	50%, 1 +	D, 2 +	D, 1 +
4	BSS	2	3	S: D, 2 + E: D, 2 +	S: F, 1 + E: D, 3 +	S: − E: D, 2 +	−	S: 20%, 1 + E: D, 2 +	S: − E: D, 3 +	S: D, 1 + E: D, 3 +	S: D, 2 + E: −	S: D, 2 + E: D, 2 +
5	BSS	3	10	S: D, 2 + E: D, 1 +	S: 20%, 1 + E: 20%, 1 +	S: F, 1 + E: D, 1 +	−	S: 20%, 1 + E: D, 1 +	S: − E: D, 3 +	S: 20%, 1 + E: D, 3 +	S: D, 2 + E: −	S: D, 2 + E: D, 2 +
6	BSS	2	2	S: D, 2 + E: D, 2 +	S: F, 1 + E: D, 2 +	S: F, 2 + E: D, 2 +	−	S: 30%, 1 + E: D, 1 +	S: F, 1 + E: D, 3 +	−	S: F, 1 + E: −	S: D, 3 + E: D, 3 +
7	MFSS	2	3	D, 3 +	−	−	−	D, 2 +	F, 2 +	20%, 2 +	D, 2 +	D, 1 +
8	BSS	2	7	S: D, 1 + E: D, 1 +	S: F, 1 + E: F, 1 +	S: F, 1 + E: D, 1 +	−	S: D, 1 + E: D, 1 +	S: F, 1 + E: D, 2 +	S: 20%, 1 + E: D, 2 +	S: D, 1 + E: F, 1 +	S: D, 2 + E: D, 2 +
9	MFSS	2	9	D, 1 +	D, 1 +	−	−	F, 1 +	F, 3 +	D, 2 +	D, 2 +	D, 2 +

*SS* synovial sarcoma, *MFSS* monophasic fibrous synovial sarcoma, *BSS* biphasic synovial sarcoma, *PDSS* poorly differentiated synovial sarcoma, *FNCLCC grading system, Fédération Nationale des Centres de Lutte contre le Cancer; *HPF* high power field, *ND* not done, *F* focal, *D* diffuse; “1 + ”, weak positivity; “2 + ”, moderate positivity; “3 + ”, strong positivity; “−”, negative; *S* spindle tumor cell, *E* epithelial tumor cell.

### Immunohistochemical features

In addition to the traditional immunohistochemical markers that are applied for diagnosing SS, we also explored the diagnostic value of a panel of markers that have recently been used in other soft tissue tumors, such as SOX-2, PAX-7, NKX3.1 and INI-1^6,16–19^. The immunohistochemical features of nine cases are summarized in Table 3. TLE-1 and Bcl-2 were diffusely positive in all subtypes of SS (9/9) (Figs. 1b, 2b). Of note, SOX-2 was positive in eight cases and three subtypes of SS (8/9). Additionally, focal immunostaining for SOX-2 was observed in MFSS (2/4), PDSS (1/1) and the spindle cell component of BSS (3/4), whereas diffuse immunostaining was observed in the epithelial component of BSS (2/4) (Figs. 1c, 2c, 4b). Unlike SOX-2, PAX-7 expression was more frequent and extensive in the epithelial component than in the spindle component of BSS (4/4) (Figs. 1d, 2d). Interestingly, weak to absent immunostaining for INI-1 was observed in MFSS (2/3, one MFSS case not analysed), PDSS (1/1) and spindle cell component of BSS (3/4) (Figs. 1e, 2e, 4c). Moreover, CD99 was diffusely positive in MFSS (4/4), PDSS (1/1), and the spindle cell component of BSS (3/4), whereas it was negative or focally positive in the epithelial cell component of BSS (4/4). CK-pan and EMA were regularly and diffusely expressed in the epithelial cell component of BSS (Figs. 2f, 3b). Furthermore, CK-pan was usually negative or focally positive (Fig. 1f), whereas EMA often demonstrated a broader positivity in MFSS and the spindle cell component of BSS. NKX3.1 was entirely negative in all subtypes of SS (9/9).

### FISH features

**Figure 5:**
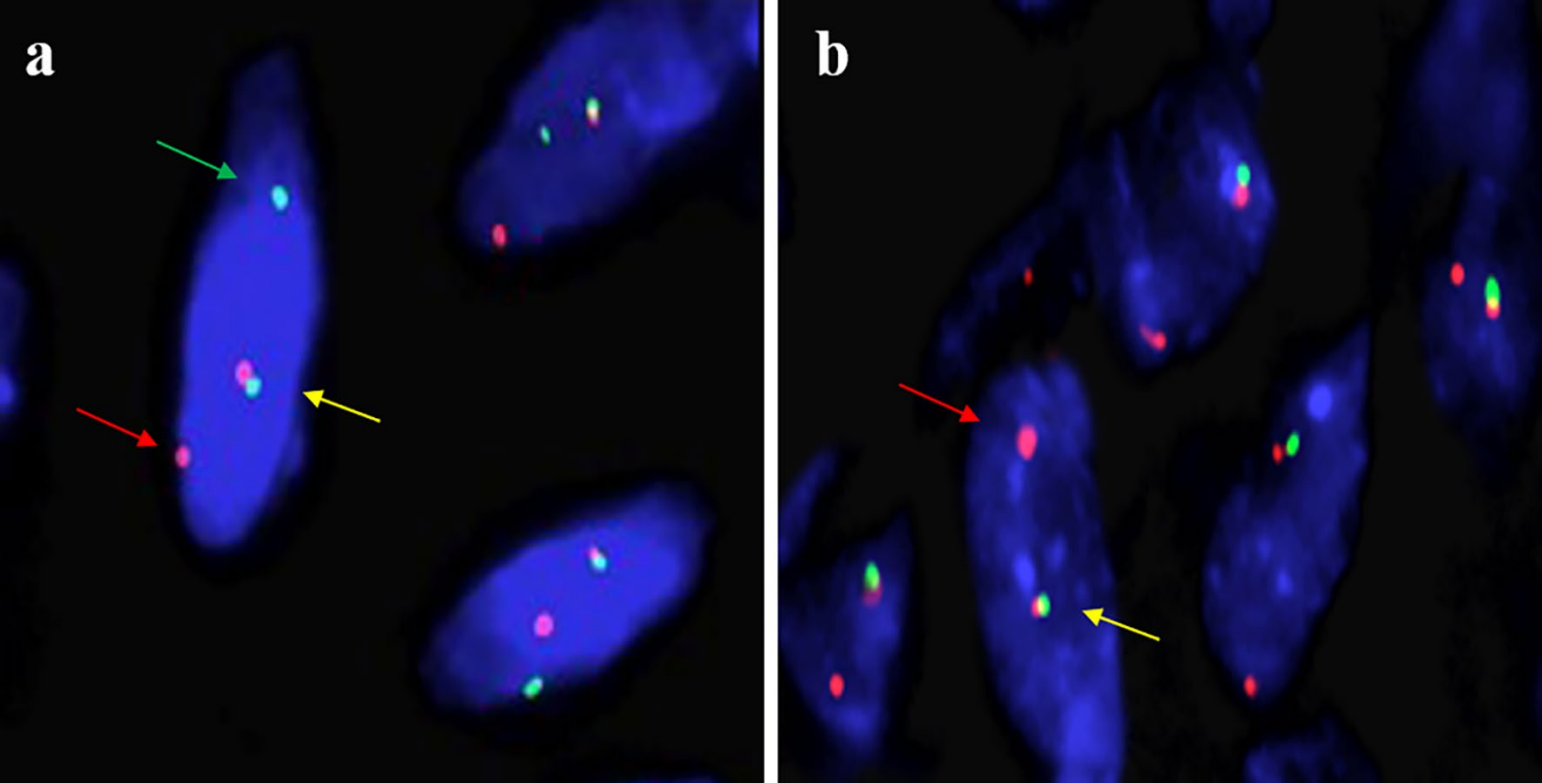
Representative FISH images of the *SS18* break-apart probe in synovial sarcoma. (**a**) In case 1, one fused signal (yellow arrowhead) and two break-apart red and green signals (red and green arrowheads) are observed, which demonstrates the *SS18* gene rearrangement. (**b**) In case 2, one fused signal (yellow arrowhead) and one isolated red signal (red arrowhead) are observed, which demonstrates the loss of the green signal.

**Figure 6:**
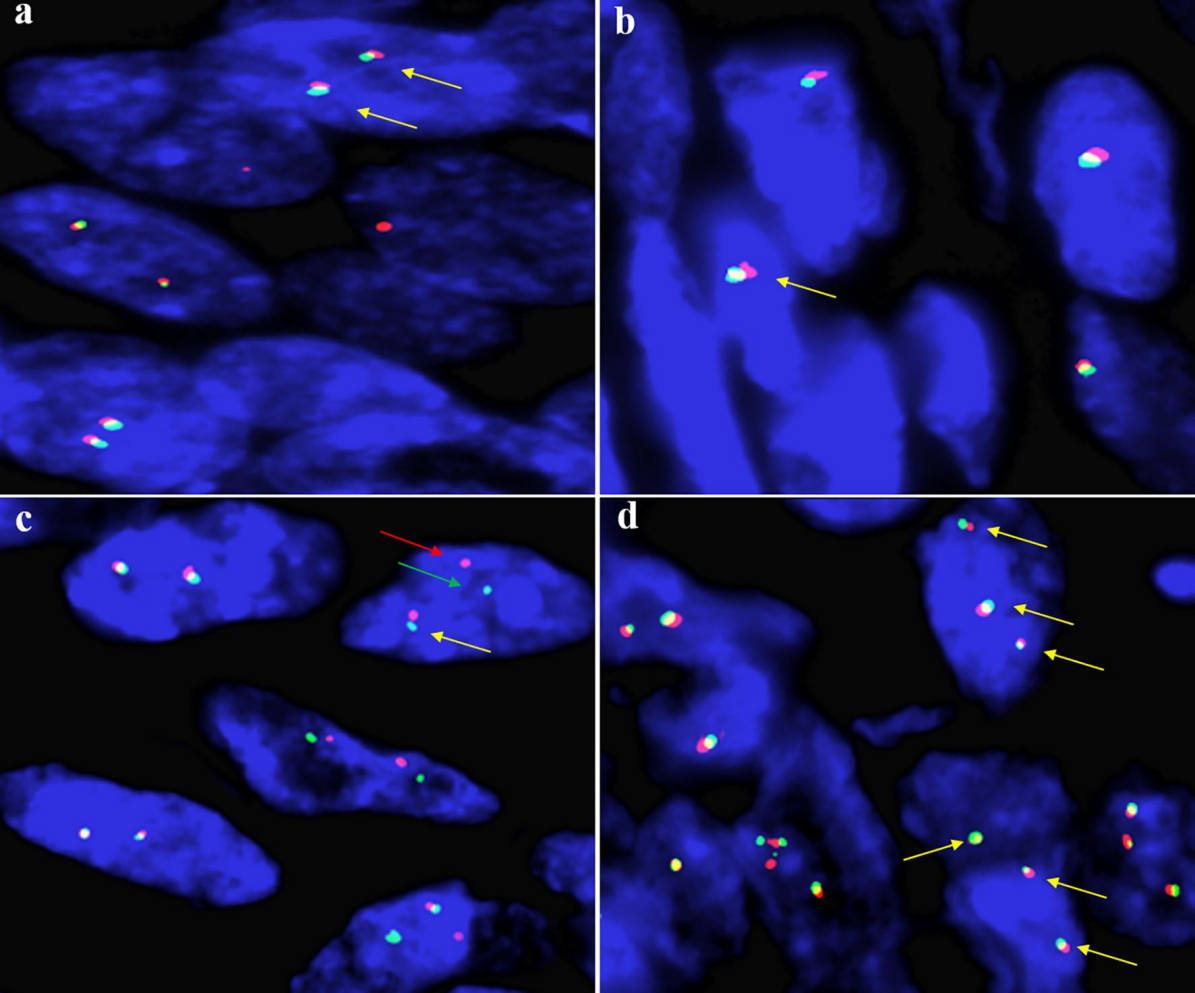
Representative FISH images of the *EWSR-1* break-apart probe in synovial sarcoma. (**a**) In case 1, two fused signals (yellow arrowhead) are observed in 94% of tumor cells, thus demonstrating no *EWSR-1* gene rearrangement. (**b**) In case 4, one fused signal (yellow arrowhead) is observed in 77% of tumor cells, which is compatible with the monoallelic loss of the *EWSR-1* gene. (**c**) In case 6, one fused signal (yellow arrowheads) and the split of red and green signals (red and green arrowhead) are found in 21% of tumor cells, thus indicating *EWSR-1* gene rearrangement. (**d**) In case 8, multiple fused signals (yellow arrowhead) are seen in 75% of tumor cells, thus indicating *EWSR-1* gene amplification.

When considering that SS had overlapping morphology and immunohistochemistry with EWSR-1 translocation-related soft tissue tumor, *SS18* and *EWSR-1* gene arrangements were detected for differential diagnosis. The details of the FISH signal pattern with the *SS18* and *EWSR-1* break-apart probes are listed in Tables 4 and 5. By using the SS18 break-apart probe for FISH detection of these cases, we observed the classical red and green break-apart signal and fusion signal (1F/1R/1G), which demonstrated the *SS18* gene arrangement in 8 out of 9 cases (Fig. 5a). Notably, 83% of tumor cells showed one fusion signal and one red signal (1F/1R) accompanied by the complete loss of the green signal in case 2 (Fig. 5b). The complete loss of green signal was unusual and was classified as representing an atypical FISH signal pattern, thus making it difficult for us to identify the *SS18* gene arrangement in case 2. For the detection of *EWSR-1* gene arrangement, no gene alteration was observed in 6 out of 9 cases (Fig. 6a). Unexpectedly, EWSR-1 gene monoallelic loss, EWSR-1 translocation and *EWSR-1* amplification were observed in case 4, case 6 and case 8, respectively (Fig. 6b–d). These data indicate that *EWSR-1* gene alteration is occasionally accompanied by *SS18* gene arrangement in SS. Therefore, the simultaneous detection of the *SS18* and *EWSR-1* genes seems essential for the differential diagnosis of SS.

**Table 4:** The molecular and cytogenetic features of SS18-SSX fusion gene in nine patients who were genetically diagnosed as SS.

Case	*SS18* break apart probe FISH signal (percent)	Fusion transcript	Gene Fusion site	RT-PCR product (bp)
1	1F/1R/1G (95%), 2F (5%)	SS18-SSX2	*SS18*: exon 10 (codon 410); *SSX2*: exon 6 (codon 111)	111
2	1F/1R (83%), 2F (17%)	SS18-SSX1	*SS18*: exon 10 (codon 404); *SSX1*: exon 7 (codon 119)	92
3	1F/1R/1G (70%), 1F/1R (9%), 1F/1G (13%), 2F (8%)	SS18-SSX1	*SS18*: exon 10 (codon 410); *SSX1*: exon 6 (codon 111)	111
4	1F/1R/1G (30%), 1F/1R (28%), 1F/1G (30%), 2F (12%)	SS18-SSX1	*SS18*: exon 10 (codon 410); *SSX1*: exon 6 (codon 111)	111
5	1F/1R/1G (40%), IF/1R (24%), 1F/1G (26%), 2F (10%)	SS18-SSX1	*SS18*: exon 10 (codon 410); *SSX1*: exon 6 (codon 111)	108
6	1F/1R/1G (44%), 1F/1R (22%), 1F/1G (28%), 2F (6%)	SS18-SSX1	*SS18*: exon 10 (codon 410); *SSX1*: exon 6 (codon 111)	111
7	1F/1R/1G (72%), 1F/1R (4%), 1F/1G (8%), 2F (16%)	SS18-SSX1	*SS18*: exon 10 (codon 410); *SSX1*: exon 6 (codon 111)	111
8	1F/1R/1G (60%), 1F/1R (20%), 1F/1G (14%), 2F (6%)	SS18-SSX1	*SS18*: exon 10 (codon 410); *SSX1*: exon 6 (codon 111)	111
9	1F/1R/1F (60%), 1F/1R (10%), 1F/1G (20%), 2F (10%)	SS18-SSX2	*SS18*: exon 10 (codon 410); *SSX2*: exon 6 (codon 111)	111

*SS* synovial sarcoma, *FISH* Fluorescence in situ hybridization, *F* fusion signaling, *R* red signaling, *G* green signaling, NCBI Reference Sequence: NM_001007559.3 (*SS18* transcript), NM_001278691.2 (*SSX1* transcript) and NM_003147.6 (*SSX2* transcript).

**Table 5:** The molecular features of *EWSR-1* gene by FISH assay in nine patients who were genetically diagnosed as SS.

Case	*EWSR-1* break apart probe FISH signal (percent)	EWSR-1 gene alteration
1	1F/1R/1G (6%), 2F (94%)	No
2	1F/1R/1G (3%), 2F (97%)	No
3	1F/1R/1G (8%), 1F/1R (3%), 1F/1G (3%), 2F (86%)	No
4	1F/1R/1G (4%), 1R/1G (8%), 1F (77%), 2F (11%)	Monoallelic loss
5	1F/1R/1G (7%), 2F (93%)	No
6	1F/1R/1G (21%), 1F/1R (6%), 1F/1G (10%), 1F (8%), 2F (55%)	Translocation
7	1F/1R/1G (4%), 1F/1R (3%), 1F/1G (2%), 2F (91%)	No
8	1F/1R/1G (7%), 2F (16%), 3F (75%), 4F (2%)	Amplification
9	1F/1R/1F (12%), 1F/1R (1%), 1F/1G (3%), 2F (84%)	No

*SS* synovial sarcoma, *FISH* Fluorescence in situ hybridization, *F* fusion signaling, *R* red signaling, *G* green signaling.

### RT-PCR and sequencing

**Figure 7:**
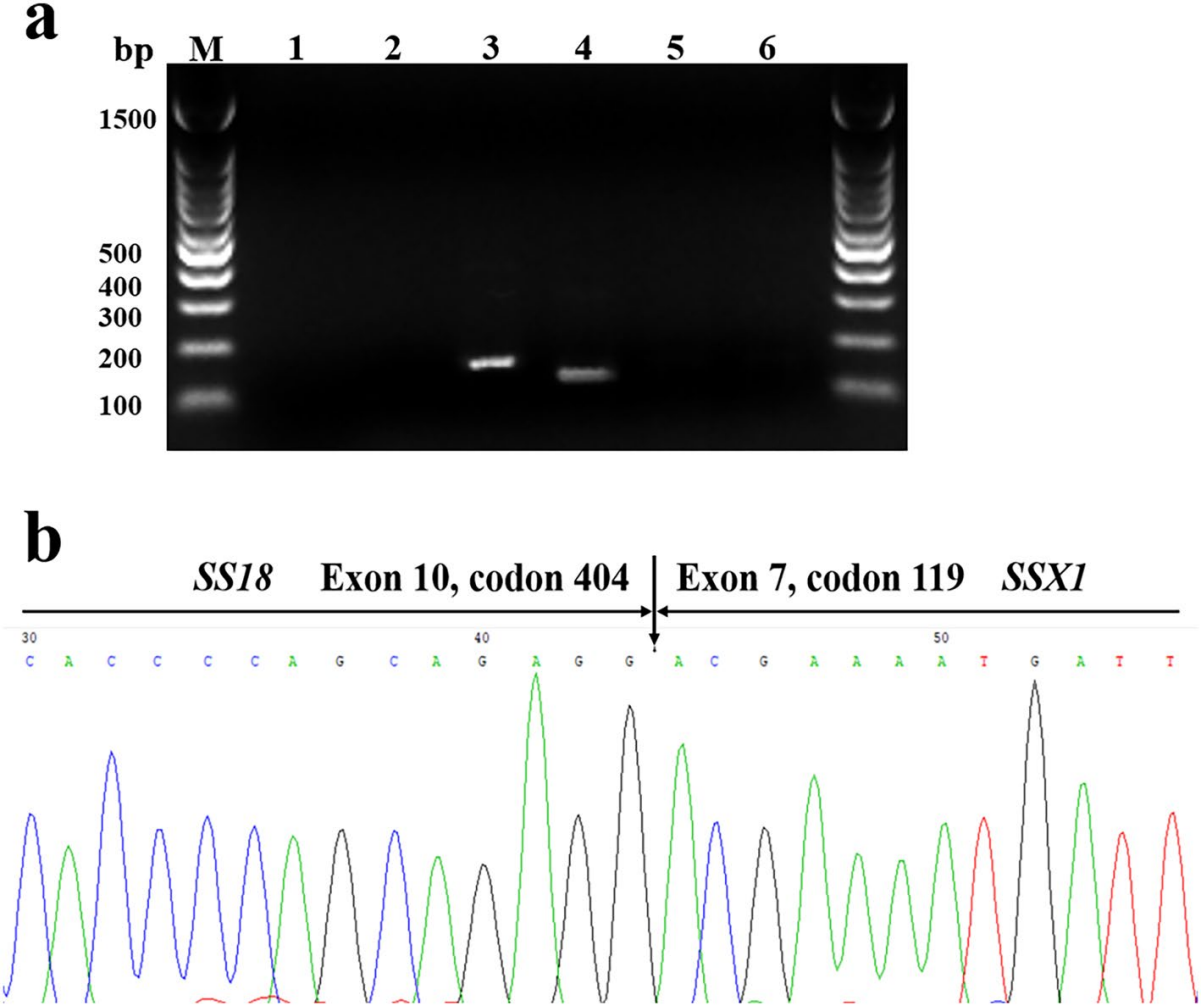
RT-PCR and nucleotide sequence analysis of the *SS18-SSX* fusion gene in case 2. (**a**) RT-PCR products were analysed via gel electrophoresis. The whole gel was represented, and its margin was cropped. Meanwhile, the raw gel was uploaded as supplementary information (Supplementary Fig. 1). Lane M, molecular size; Lane 1, blank control; Lane 2, negative control; Lane 3, GAPDH reference gene (138 bp); Lane 4, *SS18-SSX1* fusion gene (92 bp); Lane 5, *SS18-SSX2* fusion gene; Lane 6, *SS18-SSX4* fusion gene. (**b**) Sequence analysis of cDNA from the *SS18-SSX* fusion transcript in case 2. The rare fusion site (arrowhead) for the *SS18-SSX1* fusion transcript is involved in exon 10 of the *SS18* gene (codon 404) and exon 7 of the *SSX1* gene (codon 119).

We detected the *SS18-SSX* fusion gene in all cases, including the *SS18-SSX1* fusion gene in 7 cases and the *SS18-SSX2* fusion gene in 2 cases (Table 4). Subsequent cDNA sequencing demonstrated that the gene fusion site in 8 out of 9 cases involved exon 10 codon 410 in *SS18* and exon 6 codon 111 in *SSX1 or SSX2*, which is the typical fusion site (as has been previously reported)^14^*.* Notably, the *SS18-SSX1* fusion gene was unequivocally detected in case 2, which showed a complete loss of the green FISH signal (Fig. 7a). Furthermore, cDNA sequencing demonstrated that the gene fusion site involved exon 10 codon 404 in *SS18* and exon 7 codon 119 in *SSX1* in case 2 (Fig. 7b). The product length of the fusion gene was 92 bp in case 2, which was obviously shorter than that in the other SS cases (Table 4). Furthermore, the missing fragment in case 2 may be involved in the binding sites of the FISH probe labelled with green signal, explaining the possible reason for the complete loss of the green FISH signal. Consequently, a novel *SS18-SSX1* fusion site that was not previously reported was identified in case 2.

## Discussion

In this study, we first analysed the clinicopathological features and morphology subtypes in nine SS cases. The details of the clinical information are listed in Table 2. Histologically, 4 cases were classified as MFSS with uniform and delicate spindle tumor cells, as well as fascicle or dense sheet patterns, and 4 cases were identified as BSS with mixed spindle and epithelial tumor cells, as well as an occasionally vague borderline between spindle and epithelial tumor cells. Moreover, PDSS with consistently large epithelial tumor cells was identified in case 3.

SOX-2 is a transcription factor that is essential for maintaining embryonic and neural stem cells and has been documented to be a marker for cancer stem cells in various cancer types, such as squamous cell carcinoma, pancreatic cancer, breast cancer, glioblastoma, colorectal cancer and prostate cancer^20,21^. Although SOX-2 has been reported to be expressed in 58% of SS cases, the relationship between histological subtype and SOX-2 expression remains unknown^16^. PAX-7 is transcriptionally required for the specific development of skeletal muscle stem cells and has been proven to be expressed in rhabdomyosarcoma, Ewing sarcoma and PDSS; however, the expression of PAX-7 remains unclear in other histological subtypes of SS^17,22^. In addition to the traditional immunostaining markers TLE-1, Bcl-2, CD99, CK-pan and EMA, it has been demonstrated that PAX-7 expression was more frequent and extensive in the epithelial component of BSS than in that of MFSS, thus implying its significance for assisting in identifying the epithelial component of BSS in confusing cases. SOX-2 expression was observed in all subtypes of SS (88.9%, 8/9). In accordance with previous studies, INI-1 immunostaining demonstrated weak to absent expression in the majority of SS cases in this study^6,7^. With respect to NKX3.1, which was implicated in EWSR1-NFATC2 sarcoma and mesenchymal chondrosarcoma, no expression of NKX3.1 was observed in our series of cases^18,19^.

A previous study demonstrated that partial loss of green signalling was observed as an atypical FISH for the *SS18* break-apart probe^23^, whereas we observed a complete loss of green signalling in case 2. The novel atypical FISH pattern (case 2) was further verified as a *SS18-SSX1* fusion gene by using RT-PCR. Subsequently, a novel gene fusion site involved exon 10 codon 404 in SS18 and exon 7 codon 119 in SSX1 was discovered in case 2. The other cases in our series showed a typical FISH pattern for the *SS18* break-apart probe and common fusion gene site. The sequencing assay demonstrated that the fusion gene product length in case 2 was shorter than that in the other cases. We speculated that the missing product fragment included the site to which the green probe may bind, thus resulting in the complete loss of the green signal in case 2. Due to the fact that the FISH probe information was confidential, we could not further determine the reason for the complete loss of the green signal.

To explore the accompanying gene change, *EWSR-1* gene arrangement was selected for detection via the FISH assay. Unexpectedly, EWSR-1 gene monoallelic loss (1/9, case 4), translocation (1/9, case 6) and amplification (1/9, case 8) were discovered in our case series. Notably, the *EWSR-1*-translocated case possessing morphologic subtype that was BSS exhibited an ambiguous transition between epithelial and spindle tumor cells, which was different from other BSS cases. EMA immunostaining was negative in the *EWSR-1*-translocated case, but positive in the other eight cases. However, the details of *EWSR-1* translocation still need to be further investigated via sequencing. Previous studies have also demonstrated *EWSR-1* gene changes in SS, including monoallelic losses of *EWSR-1*, *EWSR-1-NR4A3* and *EWSR1-SSX1* gene fusion^15,24–26^. Therefore, the misinterpretation of the change in the *EWSR-1* gene may be a pitfall in diagnosing SS.

## Conclusions

In conclusion, a panel of SOX-2, PAX-7, INI-1 and NKX3.1 immunohistochemical markers (combined with classical markers, such as TLE-1, CK-pan, EMA, CD99 and BCL-2) can be used as an ancillary tool for the differential diagnosis of SS. Due to the atypical FISH pattern for the *SS18* break-apart probe and *EWSR-1* gene change which occasionally occur in SS, SS18-SSX gene sequencing analysis was obligatory for a precise diagnosis of SS when dealing with the above mentioned situation.

## Supplementary Information

Supplementary Figure 1.

Supplementary Table 1.

## Data Availability

The raw picture from DNA gel electrophoresis in case 2 was provided as supplementary information (Supplementary Fig. 1). The sequence data of *SS18-SSX* fusion site in each SS case were < 200 bp and not suitable for uploading the INSDC database, so the data were also provided as supplementary information (Supplementary Table 1). The datasets, not otherwise specified, used and/or analyzed during the current study available from the corresponding author on reasonable request.

## Abbreviations

SS: Synovial sarcoma
MFSS: Monophasic fibrous synovial sarcoma
BSS: Biphasic synovial sarcoma
PDSS: Poorly differentiated synovial sarcoma
FISH: Fluorescence in situ hybridization
FFPE: Formalin-fixed and paraffin-embedded
H&E: Haematoxylin and eosin
SSC: Saline sodium citrate
NP40: Nonyl phenoxypolyethoxylethanol
DAPI: 4′6′-Diamino-2-phenylindole
