# Supplementary figures and images for: Novel characteristics for immunophenotype, FISH pattern and molecular cytogenetics in synovial sarcoma
Source: Sci Rep. 2023 May 16;13:7954. doi: 10.1038/s41598-023-34983-2 (PMC10188594; doi:10.1038/s41598-023-34983-2)

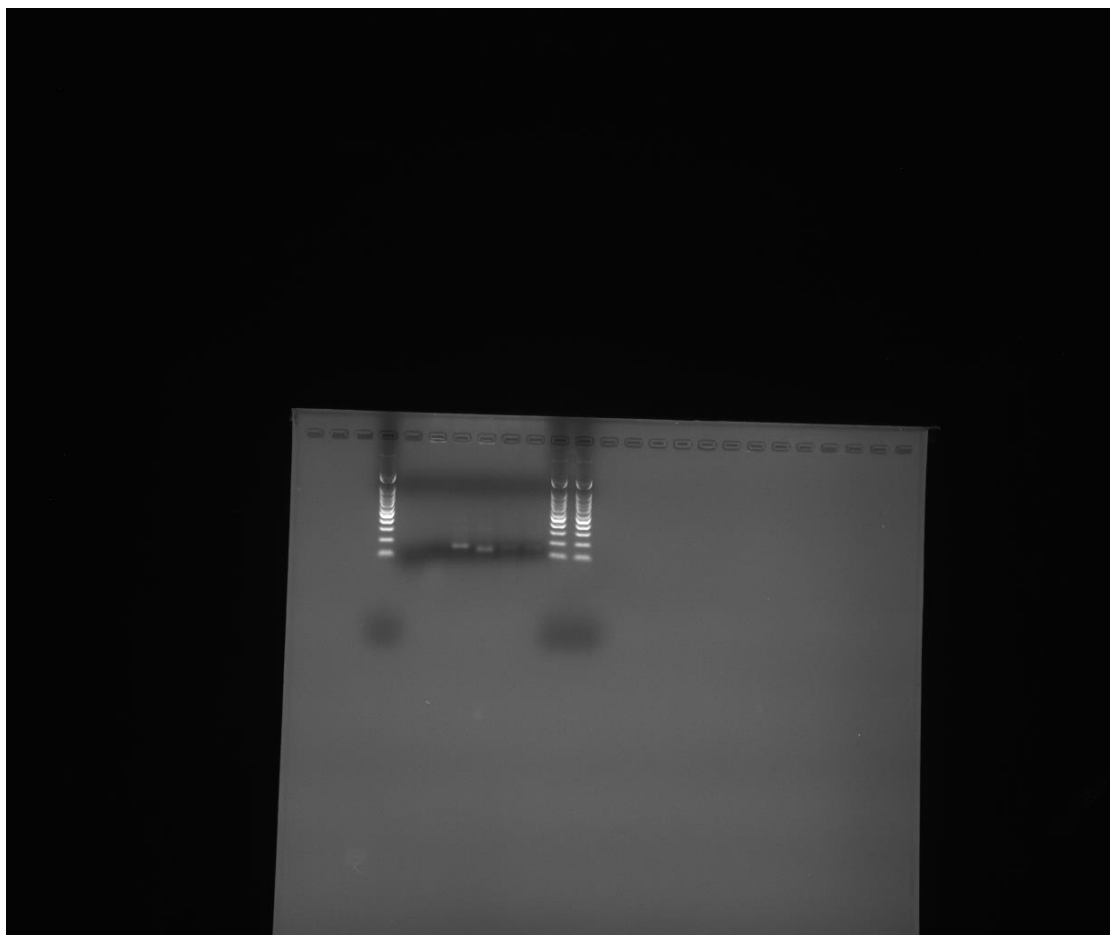

Supplementary Figure 1. This is the raw gel photo for the figure 7a in case 2.

Supplement: Supplementary file 1 — Supplementary Figure 1. [file 41598_2023_34983_MOESM1_ESM.pdf]
